# INCT in oncogenetics focusing on hereditary breast-colorectal carcinoma syndrome

**DOI:** 10.1186/1753-6561-7-S2-K19

**Published:** 2013-04-04

**Authors:** Silvia Regina Rogatto

**Affiliations:** 1 Faculty of Medicine, São Paulo State University, UNESP, Botucatu, SP, Brazil; 2 International Center of Research and Training (CIPE), Hospital A.C. Camargo, São Paulo, SP, Brazil

## 

The National Institute of Science and Technology in Oncogenomics (INCT) established a scientific program that links 46 researchers and supports their efforts to translate research findings into clinical practice, including clinical trials. Our initiative focused in the management of resources of three areas: research networks; chair programs and infrastructure support; transference of new knowledge, which can reduce the burden of cancer, quickly and efficiently to the Brazilian healthcare system. The National Institute of Science and Technology in Oncogenomics was created by the Brazilian Ministry of Science and Technology in search of excellence in scientific activities at an international level, by means of programs and instruments made operationally by CNPq (National Council for Scientific and Technological Development) and FAPESP (São Paulo Research Foundation).

The major aim of this study is recruit cancer patients from families with a history of family aggregation or hereditary cancer and relatives, affected or not, in order to obtain molecular and epidemiological data. We are investigating genomic alterations in the probands and in their relatives, including at least one who is a cancer carrier (not necessarily the same tumor type). Genomes vary from one another in several ways and the totality of this genetic variation is the basis of human traits heritability. Genome re-sequencing studies have shown that the bases that vary among genomes reside in CNVs (Copy Number Variations) ranging in size from kilobases (kb) to megabases (Mb), which are not identifiable by conventional chromosomal banding. Deletions, duplications, amplifications, insertions, and translocations can result in CNVs. In addition, balanced genomic inversions leading to DNA structural variations that do not cause CNV can nevertheless contribute significantly to genome instability. Despite extensive studies, the total number, position, size, gene content, and population distribution of CNVs remain elusive. Although it is estimated that CNVs may account in a large proportion of the human genome, the association between CNVs and human cancer is limited. It is anticipated that the application of array CGH techniques and next-generation sequencing will reveal a significantly larger scale of structural variation among different individuals and populations, as the majority of CNVs appear smaller in scale and are beyond the resolving capability of current arrays [1,2]. CNVs can be inherited or sporadic; large de novo CNVs are thought more likely to be disease causative. However, the phenotypic effects of CNVs are sometimes unclear and depend mainly on whether dosage-sensitive genes or regulatory sequences are affected by the genomic rearrangement. Sporadic disease can also result from a combination of two CNVs at a single locus or theoretically from two or more CNVs at different loci from two normal parents [3].

Despite the fact that cancer is an acquired disease caused by various factors, there is clear evidence that inherited factors play a significant role. Some of these inherited factors represent loss-of-function mutations in tumor suppressor genes, resulting in a high relative cancer risk among carriers. In particular, rare constitutional CNVs may affect important cancer-associated genes or pathways, providing an explanation for high-risk cancer families. The first approach of this study is to investigate clinically and genetically the most common cancers associated with hereditary predisposition (breast, ovary, colorectal, head and neck carcinomas) in a Brazilian Network of Cancer involving Reference Health Care Cancer Centers.

It is accepted that 5 to 10% of all cancers are hereditary or familial [4]. The majority of hereditary neoplasias related to breast cancer are associated with germline mutations in *BRCA1* and *BRCA2*. However, inherited mutations related to other genes and/or related to certain syndromes also influence the increased risk of developing cancer. Li-Fraumeni syndrome results in a mutation on gene *TP53* and is related to increased risk of developing tumors at a young age. A deletion in gene *CHEK2* is associated with a two-fold greater risk of the patient presenting breast cancer. Hereditary nonpolyposis colorectal cancer (HNPCC) associated with mutation in the DNA damage repair genes, such as *MLH1* and *MSH2*, constitutes a risk factor for the development of extracolonic tumors, including breast tumors [5].

The possibility of identifying a Brazilian profile of the syndrome has permitted our group to propose new tracing strategies aimed at contributing to early detection of disease carriers among Brazilians. The Department of Oncogenetics of the AC Camargo Hospital was created in 2000 and since then, more than 4,500 patients and family members have received Genetic Counseling. The Oncotree software is used to monitor family history data. In the AC Carmago Hospital peripheral blood DNA samples of patients with hereditary cancer or family history or familial aggregation of cancer have been recruited over the years. Some of these families were selected because they presented all but one of the international criteria adopted in the categorization of a Familial Cancer Syndrome. In this project, affected family members (with screening of pathogenic mutations in the major candidate genes) are under evaluation by array-CGH and next generation sequencing. The objective is to publicize rare genomic alterations that could contain new hereditary predisposition genes, aimed at defining or identifying new markers of risk of susceptibility to cancer. Here, we discuss the new findings in probands without mutations in genes frequently described as associated with the breast and ovary syndrome, Lynch syndrome, breast and colorectal syndrome as well as future perspectives of application of these results.

## Competing interests

There are no competing interests in this presentation.
